# Effects of Social Media Use on Youth and Adolescent Mental Health: A Scoping Review of Reviews

**DOI:** 10.3390/bs15050574

**Published:** 2025-04-24

**Authors:** Nadine Agyapong-Opoku, Felix Agyapong-Opoku, Andrew J. Greenshaw

**Affiliations:** 1 Department of Psychiatry, University of Alberta, Edmonton, AB T6G 2R3, Canada; f.agyapong-opoku1@universityofgalway.ie (F.A.-O.); andy.greenshaw@ualberta.ca (A.J.G.); 2 College of Health Sciences, University of Ghana Medical School, Accra P.O. Box GP 4236, Ghana; 3 School of Medicine, University of Galway, H91 TK33 Galway, Ireland

**Keywords:** youth, adolescent, mental health, social media, review

## Abstract

**Background:** The impact of social media on adolescent mental health has become a critical area of research as social media usage has surged among youth. Despite extensive research, findings on this relationship remain inconsistent, with various studies reporting both negative and positive effects. This scoping review aims to clarify the multifaceted nature of this relationship by analyzing the recent literature. **Objective:** This review aims to analyze the current evidence regarding the effects of social media use on adolescent mental health, identify consistent patterns and discrepancies in the findings, identify gaps in our knowledge, and highlight opportunities for further research. **Methods:** A scoping review was conducted following Arksey and O’Malley’s five-stage approach. Searches were performed in PubMed, MEDLINE, Web of Science, and Scopus for articles published between July 2020 and July 2024. Inclusion criteria were systematic reviews, umbrella reviews, narrative reviews, and meta-analyses written in English focusing on youth/adolescents’ mental health and social media. The search strategy identified 1005 articles, of which 43 relevant articles survived the reviewer selection process, from which data were extracted and analyzed to inform this review. **Results:** The majority of studies linked social media use to adverse mental health outcomes, particularly depression and anxiety. However, the relationship was complex, with evidence suggesting that problematic use and passive consumption of social media were most strongly associated with adverse effects. In contrast, some studies highlighted positive aspects, including enhanced social support and reduced isolation. The mental health impact of social media use, specifically during the COVID-19 pandemic, was mixed, with the full range of neutral, negative, and positive effects reported. **Conclusions:** The nature of social media’s impact on adolescent mental health is highly individualistic and influenced by moderating factors. This review supports the notion that social media’s effects on adolescent mental health can be context specific and may be shaped by patterns of usage. A focus on longitudinal studies in future research will be useful for us to understand long-term effects and develop targeted interventions in this context. Enhancing digital literacy and creating supportive online environments are essential to maximizing the benefits of social media while mitigating its risks.

## 1. Introduction

Since the inception of social media, the relationship between social media and mental health remains a heavy area of contention, and social media usage and mental illness have both become increasingly prominent amongst youth and adolescents. Research demonstrates that more than 90% of adolescents possess at least one social media account, and this percentage shows no indication of declining (Kucharczuk et al., 2022). A systematic review and content analysis reported that in 2022, TikTok engagement among youth and adolescents increased the most globally among other existing social media platforms (McCashin & Murphy, 2023). Other popular social media platforms adolescents frequently engage with include Instagram and YouTube (Bozzola et al., 2022). The nature of social media applications renders them particularly enticing for adolescents at this vulnerable stage of development, as the socio-affective circuitry of the brain is rapidly developing. This may increase sensitivity to social information, the desire for social rewards, and anxiety about peer appraisal (Somerville, 2013).

Recent findings suggest that the mental health of adolescents is substantially influenced by their social media use (Weigle & Shafi, 2024); however, the association between social media use and mental health is highly complicated and multifaceted (O’Reilly, 2020). Mental illness accounts for over 12% of diseases globally and 23% in developed countries (Xavier et al., 2013). Specific to children and adolescents, 10–20% are universally affected by mental illness (Kieling et al., 2011). It is an internationally accepted notion that a relationship exists between social media and mental health; however, the exact nature of this relationship is still subject to ongoing research. There is a wide range of literature that highlights this uncertainty, with each study directly examining or addressing the relationship but collectively drawing inconsistent conclusions (Haddad et al., 2021; Liu et al., 2022; Sedgwick et al., 2019).

Results of a longitudinal study conducted among adolescents in the U.K. suggested a trend for a relationship of increased time on social media and diminished mental health (Plackett et al., 2023), although such an association does not necessarily imply cause–effect (Bozzola et al., 2022). Other studies have affirmed, through varied mechanisms, a significant and direct association between social media use in adolescents and increased depression, suicidal ideation, body dissatisfaction, anxiety, and other mental-health-related issues (Jiotsa et al., 2021; Lin et al., 2016; Vidal et al., 2020; Wang et al., 2022). On the other hand, some evidence also indicates that social media may act as a positive platform that confers therapeutic benefits to individuals experiencing mental health issues by fostering healthy and supportive online communities, thereby preventing further deterioration of their mental health (Ulvi et al., 2022). This notion is consistent with other findings stating that the use of social media is a positive asset for mental health in that it boosts quality of life, social support, and wellbeing and reduces stress. Notably, some evidence indicates that for each hour of increase in social media use among adolescents, there was a 13% increased risk of depression, an association which was more robust for adolescent females than males (Liu et al., 2022). It is important to note that the quality rather than the quantity of social media use appears to be a crucial determining factor for the valency of this exposure and the risk/benefit relationship (Davila et al., 2012). In more specific terminology, problematic use of social media is what renders it a negative entity for adolescent mental health (Shannon et al., 2022).

Several conflicting theories exist regarding the relationship between mental health and social media. In light of this background information, we review and summarize this relationship with current evidence and highlight variables that lead to the diversity of research conclusions in the literature. It is important to note that our analysis examined evidence for effects of social media use per se; it did not focus on any interventions that may have been presented in the context of interactions with social media. 

## 2. Methodology

### 2.1. Study Design

The design for this review of reviews aligns with Arksey and O’Malley’s five-stage approach to scoping reviews (Arksey & O’Malley, 2005): (1) develop the research question; (2) identify relevant studies; (3) select articles; (4) chart the data; and (5) summarize and report the results.

### 2.2. Identifying the Research Questions

This scoping review’s objective is to examine the scope of what is known about the impact on mental health outcomes in adolescents who use social media.

### 2.3. Identifying Relevant Studies

On 23 July 2024, a systematic literature search was conducted via electronic bibliographic databases, including PubMed (Public/Publisher MEDLINE (NLM journal articles database)), MEDLINE (Medical Literature Analysis and Retrieval System Online), Web of Science, and Scopus Elsevier. The search was conducted using Boolean operators ‘OR’/‘AND’ between search terms. The specific search terms were (‘Social media’ or ‘Social networks’ or ‘Social platforms’ or ‘Digital media’ or ‘Social media platforms’ or ‘Social media networks’ or ‘Social media sites’ or ‘Social media apps’ or ‘Social networking apps’) AND (‘Adolescent’ or ‘young adult’ or ‘Youth’ or ‘Teenager’ or ‘Youngster’ Or ‘Minor’ or ‘Young person’) AND (‘Mental health’ or ‘Psychological health’ or ‘Psychological wellbeing or ‘Mental wellbeing’ or ‘Emotional stability’ or ‘Mental wellness’). Inclusion criteria were systematic reviews, umbrella reviews, narrative reviews, and meta-analyses written in English focusing on youth/adolescents’ mental health and social media. Excluded studies included gray literature, dissertations, preprints, and conference proceedings. Publication restrictions were applied (July 2020–July 2024) to ensure that, practically, the most current review literature was captured at the time of completion of data extraction. The July 2020 publication date was chosen to capture a period of dynamic change, as that date marked the rapid rise in the number of reviews published in this domain and corresponded to the beginning of the COVID-19 pandemic, which signaled a shift towards increased digital information use. This scoping review expanded upon previous reviews of the literature. It was adopted as the current knowledge suggests several inconsistencies in prior work concerning the role of social media in the mental health of adolescents.

### 2.4. Article Selection

Articles were retrieved from several databases and extracted to be imported into Covidence, a web tool for screening the literature. Duplicates were automatically removed. Two researchers independently reviewed the citations during the title/abstract screening and the full-text review phase based on a specific eligibility criterion. All discrepancies were resolved through discussion and consensus.

Studies were included if (1) youth/adolescents were the subjects of their study, (2) they were involved in the use of social media (e.g., Snapchat, Instagram, TikTok, etc.), and (3) studies were published between July 2020 and July 2024. Articles were additionally limited to systematic reviews, umbrella reviews, narrative reviews, and meta-analyses written in English. Articles were excluded from the review if the subjects of the review had pre-existing mental health illnesses, if the study focused on the impact of mental health on social media use, or if the study addressed dimensions of health other than mental health. Articles were also omitted if the study participants were adults or children.

We identified 61 articles for full-text review but, upon closer examination, excluded 19 articles that did not meet the inclusion criteria.

### 2.5. Data Charting and Extraction

The following information was extracted from the included articles according to the following domains: author(s) name, year of publication, total number of articles, review type, aim of review, participants, sample size (N), social media application(s), bias assessment, key findings, and recommendations.

### 2.6. Collating, Summarizing, and Reporting the Results

This scoping review summarizes recent evidence regarding the mental health implications of using social media. All of the relevant data were organized into tables and validated by two team members. The characteristics and results reported in each article are summarized below.

## 3. Results

### 3.1. Article Search Results

**Figure 1:**
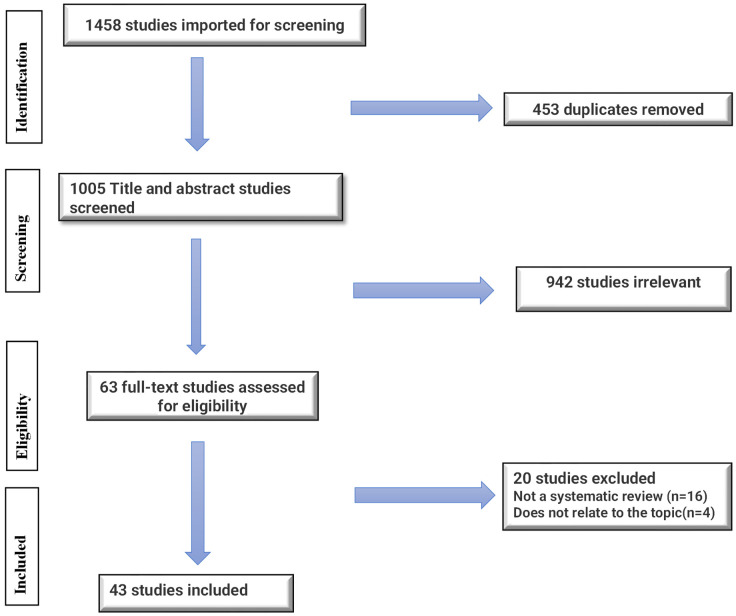
PRISMA diagram detailing the study identification and selection process. PRISMA, Preferred Reporting Items for Systematic Reviews and Meta-Analyses.

After removal of duplicates through the use of Covidence software, the search strategy yielded 1005 of 1458 studies eligible for primary screening through the search strategy in the electronic databases. From title and abstract screening of those 1005 articles, 63 studies were retained for full-text review. After full-text review, 20 studies were excluded, the primary cause being that the studies did not fall under the requirements of the review’s inclusion criteria, and 43 review articles met the inclusion criteria and were incorporated into this review. The PRISMA flow diagram (Figure 1) below outlines this in detail.

### 3.2. Overview of the Included Reviews

Overall, 12 included reviews were classified as systematic reviews, whilst another 12 reviews were classified as meta-analyses, and 2 studies were classified as both systematic reviews and meta-analyses. A multitude of other review types was also included (scoping reviews, multinational reviews, systematic reviews and meta-analyses, umbrella reviews, narrative reviews, qualitative literature reviews). The articles included in the individual studies were published from as early as 2000 to as recently as 2024.

### 3.3. Targeted Conditions

There were a variety of mental health illnesses identified, measured, and discussed in the reviews under the current search protocol. The objectives of the majority of reviews targeted the measurement of depression and/or anxiety (n = 17, 40%), suicidal ideation and/or self-harm (n = 6, 14%), and body dysmorphia (n = 5, 12%). A combination of various mental health disorders was measured and discussed in the remaining studies (n = 15, 35%).

### 3.4. Opportunities for Refinement of Research Questions and Strategic Intervention Proposals

A majority of reviews expressed the need for a larger quantity of longitudinal studies to be present in research for further analysis of the relationship between social media use and mental health outcomes. Other studies recommended a varied set of specific research questions that would be beneficial for inclusion in the existing large pool of research questions on this topic.

In terms of problematic social media use, three studies suggested the implementation of intervention strategies to reduce the impact of social media use on the mental health of individuals. Intervention mechanisms included restriction of screen time (Damodar et al., 2022) as well as prevention and intervention strategies to lessen the functional impact on individual mental health.

### 3.5. Aim of the Reviews

The general objectives of the reviews were to assess the mental health outcomes of social media use in terms of youth and adolescent mental health. Some reviews analyzed this research question during the specific duration of the COVID-19 pandemic (n = 5, 11%). One review analyzed the relationship between time spent on social media and the effect on mental health. Another study investigated the specific use of highly visual social media, while another explored the impact of problematic social media use on mental health.

### 3.6. Number of Articles

The number of articles included in each review was not always reported, but it was an average of 33 articles per review when the number of articles was specified. Table 1 gives details of the articles included in this review. 

### 3.7. Key Findings

From Table 1 above, the review found mixed results, with both negative and positive outcomes associated with social media engagement. The majority of the review articles reported that frequent use of social networking sites (S.N.S). was associated with poor mental health outcomes. Seven studies reported an association between social media and both depression and anxiety (Damodar et al., 2022; Draženović et al., 2023; Keles et al., 2020; Marciano et al., 2022; Rathod et al., 2022; Schønning et al., 2020; Tibber & Silver, 2022). Thirteen studies reported a connection between social media use and depression (Arias-de la Torre et al., 2020; Cunningham et al., 2021; Haddad et al., 2021; Khalaf et al., 2023; Liu et al., 2022; Santos et al., 2023; Sarmiento et al., 2020; Zhu et al., 2023; Lee et al., 2022; Vidal et al., 2020; Yue & Rich, 2023; Ivie et al., 2020; Piteo & Ward, 2020). One article each reported an association between social media use and depression, anxiety and stress (Shannon et al., 2022) and an associations with ADHD (Thorell et al., 2022). An association was also found between heavy social media/internet use and increased suicidal ideation or self-harm behavior (Abi-Jaoude et al., 2020; Biernesser et al., 2020; Chen et al., 2024; Sedgwick et al., 2019).

Some studies also reported an association between social media use and heightened body image concern and dissatisfaction (McCrory et al., 2020; Moss et al., 2023; Ryding & Kuss, 2020), whilst another study (Sala et al., 2024) reported social media association with several mental health issues including: (i) depressive and anxiety symptoms, (ii) problematic use and addiction, (iii) eating behaviors and body image concerns, and (iv) deliberate self-harm and suicidality are among the identified risks to which adolescents are subjected. The study by Suhag and Rauniyar (2024) focused on social media and self-image- and eating-related activities, and reported an association between social media use and less satisfaction with one’s body. It was reported by one review article that increased social media use may positively impact adolescents’ affective and cognitive empathy and broaden their ability to communicate their feelings (Primack et al., 2022). This study also reported an association between social media and several mental health issues such as anxiety, depression, schizophrenia, and autism spectrum disorders, obsessive-compulsive disorder depression, anxiety, and sleep disturbance. While many studies pointed to negative impacts of social media, Keles et al., reported mixed findings (Keles et al., 2020).

## 4. Discussion

### 4.1. Overview

This review of reviews analyzed the association between use of social media and the mental health of youth and adolescents. The results of this analysis largely affirm what is a variable associative relationship between youth and adolescent social media use and mental health. The nature of this relationship is reported as highly inconsistent between studies and highly complex and multifaceted, hence our description of a variable associative relationship, and multiple factors must be taken into consideration to disentangle the likely importance of specific contexts. Within this variable set of reports for the associative relationship, this review identified several consistent patterns between the studies, as described below.

### 4.2. Anxiety and Depression

Anxiety and depression were the two most frequently measured outcomes of social media use in adolescents. The majority of included articles in one review positively linked depression (82.6%) and anxiety (78.3%) with social media use. In 13% of articles included in the same review, a ‘dose-response’ relationship was noted between time spent on social media and depression and anxiety symptoms (Damodar et al., 2022). In several other reviews (Cunningham et al., 2021; Damodar et al., 2022; Keles et al., 2020; Lee et al., 2022; Liu et al., 2022; Piteo & Ward, 2020; Prasad et al., 2023; Sarmiento et al., 2020; Shannon et al., 2022; Vidal et al., 2020; Zhu et al., 2023), results of a similar nature were reported, adding further evidence to support these outcomes.

### 4.3. Active vs. Passive Social Media Use

Three reviews concluded that social media in and of itself does not inherently pose a threat to youth and adolescent mental health, and it is rather the *problematic* use of these applications that is harmful. These reviews articulated evidence for a significant relationship between problematic, addictive, and misuse patterns of social media use in youth and negative mental health (Piteo & Ward, 2020; Shannon et al., 2022; Vincente-Benito & del Valle Ramirez-Duran, 2023). One review indicated a higher strength of effect when considering the impact of problematic use of social media on depression as opposed to either time spent on social media or intensity of social media use (Cunningham et al., 2021). In a similar fashion, another three studies drew a clear distinction between the specific nature of social media use (active vs. passive) and mental health outcomes. Passive social media use is defined as observing content, nighttime social media consumption, and engaging in appearance-based activities, while active social media use is associated with cyberbullying or sexting (Prasad et al., 2023). This necessary distinction was observed in a study identifying that girls who made passive use of Facebook and boys who actively used Facebook were more likely to be affected by the negative impacts of Facebook (Frison & Eggermont, 2016). A similar study concluded that a cause-and-effect relationship between social media and mental health outcomes was too simplistic and that it is rather adolescent activity online (e.g., active vs. passive use), among several other factors, that defines this relationship (Sarmiento et al., 2020). It was observed that passive social media consumption had strong associations with depressive mood, social compassion, and greater body dissatisfaction, among other negative mental health implications (Course-Choi & Hammond, 2021; Ryding & Kuss, 2020), whereas active social media use was largely associated with increased wellbeing (Bottaro & Faraci, 2022). Several studies that reported the negative outcomes adolescents and youth faced upon social media use did not take into consideration the reason for the consistency of these results, which may now evidently be attributed to individual passive use of these applications. This would suggest consistency in the notion that social media use, more specifically passive social media use, is a large contributor to negative mental health outcomes among youth and adolescents.

### 4.4. Body Dissatisfaction and E.D.

Several studies affirmed increased outcomes of eating disorders and social media use (Schønning et al., 2020), attributing this result to obsessiveness with photo editing, misuse or intensive use of social media, and posting with a blurred demarcation of real vs. virtual presentations (Sharma & Vidal, 2023; Vincente-Benito & del Valle Ramirez-Duran, 2023). Fitspiration trends on social media also contribute to these negative mental health outcomes and result in the development of psychopathological symptoms in young people, including eating disorders (Cataldo et al., 2021). Several studies consistently brought forth evidence to suggest that when young people engage with social media platforms that emphasize outward attractive appearance, the result is heightened levels of stress and eating disorders (Suhag & Rauniyar, 2024). In a related fashion, studies that explored the photoshopping of images on social media and body dissatisfaction/body image in adolescents also unveiled negative implications (Sharma & Vidal, 2023; Vincente-Benito & del Valle Ramirez-Duran, 2023).

### 4.5. Suicidal Ideation

A specific relationship between suicidal ideation per se and social media use was not recognized as a prominent focus of the published literature based on our present analysis of reviews, which indicates an opportunity and need for research focus and synthesis in relation to studies in that context. However, one review identified evidence for a possible direct and independent association between excessive social media use and increased suicide attempts in seven primary research studies (Sedgwick et al., 2019). A correlation between time spent on Instagram and engagement in deliberate self-harm activities was also observed in a review by Moss and colleagues (Moss et al., 2023). It was additionally observed that total screen use is significantly associated with heightened risks of subsequent self-harm and suicidal behaviors among young people (Chen et al., 2024; Schønning et al., 2020), and the effects appeared to be greatest among girls (Abi-Jaoude et al., 2020). In contrast, social media use was not significantly associated with self-harm or depression among boys (Santos et al., 2023). Chen et al. (2024) also provided a review of further evidence supporting these conclusions, finding that total problematic screen use, such as through exposure to and expression of direct self-harm (D.S.H.) content (Biernesser et al., 2020) and cyberbullying victimization, was associated with heightened risk of the development of future self-harm and suicidal behavior in young people.

### 4.6. Positive and Negative Effects of Social Media in Light of COVID-19

In light of the pandemic being within the required time frame of studies used in this review, several studies specifically analyzed the impact of heightened social media use during this time on the mental health of young people. Studies observed a negative impact of social media use on the mental health of adolescents, with the most frequently reported mental health illnesses being anxiety and depression (Draženović et al., 2023; Lee et al., 2022); however, another concluded a negative but nonsignificant correlation between social media use and wellbeing during this time period (Wong et al., 2022). This was attributed to the diverse moderators existing in the relationship between social media and wellbeing. To specify, the study noted that the tendency to use social media as a coping mechanism during the pandemic was negatively correlated with higher levels of wellbeing, which was suggestive of the fact that individuals with a higher tendency to use social media as a form of coping had a higher likelihood of experiencing poorer emotional wellbeing than individuals who reported a lower tendency to use social media as a form of coping. It is additionally important to note that an increased risk of addictive social media use was seen in individuals suffering from greater COVID-19 stress; however, it was also observed in two studies included in a review that social media had positive implications for mental health through humorous content and positive exchange (Draženović et al., 2023). It is also important to note that not all forms of social media were negatively associated with mental health during this time. Exceptions include self-disclosure in the context of mutual online friendship, one-to-one communication, and positive and funny online experiences, which alleviated feelings of loneliness and stress (Marciano et al., 2022).

### 4.7. Positive Effects of Social Media

Social media is often primarily associated with negative implications for mental health; however, it is undeniable that some positive implications can also be observed. Three out of twenty-five studies summarized in a review by McCrory et al. (2020) noted that there was a positive relationship between social media use in terms of duration of use and the wellbeing of young people. Social media is also credited for its benefits among individuals facing social isolation in that it may reduce isolation, and it is associated with feelings of support and social connectedness (Biernesser et al., 2020). Social media is also effective in its ability to prevent depression and suicide and ensure easy access to supports (Vidal et al., 2020) and by providing a sense of community (Abi-Jaoude et al., 2020; Popat & Tarrant, 2023). One review reported evidence that adolescents who sought social support via Facebook later reported improved depressive symptoms if support was received and, contrarily, worsened symptoms if support was denied (Frison & Eggermont, 2015). The use of social media has, furthermore, enhanced traditional mental health treatment, as researchers have used freely available data from social media to conclude whether patients are exhibiting suicidal and/or psychotic behaviors (Primack et al., 2022).

### 4.8. No Effect

Although the two opposing sides of the spectrum are heavily debated in terms of the relationship between social media and mental health, there also exists a middle ground where some studies suggest a null effect. Four of the twenty-five studies reviewed by McCrory et al. (2020) suggested a null relationship between social media use and mental health among young people.

It is evident that there are numerous contextual factors in terms of ‘confounders, mediators, and moderators’ that may explain the discrepant findings presented in the sections above (Blomfield neira & Barber, 2014).

### 4.9. Limitations

Although they were necessary, many methodological restrictions were also a large source of limitation in this review. Only articles written in the English language were included for consideration. This resulted in the omission of valuable information that may have been found in reviews written in other languages. This imposes a limitation on the results of this review, as well as their interpretation. Furthermore, the overall search strategy may have been biased in that several articles were selected from health and science databases; however, relevant studies may have been potentially acquired by searching other bibliographic databases. Lastly, several new terminologies are rapidly arising concerning mental health, social media, and even age demographics. This would inevitably affect search strategies, as potential sources of research could have been missed. In addition, this review of reviews focused on peer-reviewed academic publications and did not consider the gray literature. Although this is consistent with a scientifically rigorous approach, in the very fast-moving area of digital influences on mental health, there are undoubtedly numerous insights to be gleaned from gray literature sources (Yoshida et al., 2024). Notwithstanding these limitations, this scoping review has offered relevant insight into the mental health implications of the use of social media for youth and adolescent mental health.

### 4.10. Implications

The use of social media is practically inevitable among adolescents; therefore, this review has reiterated the importance of parental involvement and educational intervention regarding their use of social media applications. It is essential for schools and educational institutions to prioritize digital literacy and mental health education in order to equip youth and adolescents with the necessary skills required for them to engage with social media. Additionally, there should be heightened availability of accessible online mental health resources for adolescents by schools and community organizations. These could include, but are not limited to, support services, such as counseling and other online resources through social media, to offer targeted support to adolescents subject to the dual nature of social media. Lastly, it would be beneficial and effective for social media technology companies and mental health professionals to collaborate in order for social media platforms to invest in the development of features that foster healthy user interactions and avenues for mental health to be discussed so as to fortify mechanisms for users to report distressing content.

## 5. Conclusions and Future Directions

This review emphasized the multi-dimensional nature of the relationship between social media and mental health. It is evident that this relationship is highly complex and, therefore, cannot be subject to a straightforward verdict. This is reiterated by the countless articles that have addressed this relationship but, when taken together, have reached inconsistent conclusions. It has become apparent through this review that the nature of social media has the potential to be molded by the individual to whom it is subject. To specify, several moderating factors exist, which have been discussed in this review, that undoubtedly impact the mental health outcomes observed when adolescents inevitably engage with social media platforms. These factors are also complex in their nature and do not have universal implications.

It is imperative for future studies to execute additional longitudinal studies in order to emphasize the long-term effects of social media use on adolescent mental health. This is essential to provide deeper insights into long-term consequences. In addition to this, rather than broadly studying the effect of social media on adolescent mental health, longitudinal studies should place restrictions on the permitted activity of adolescents on social media during the period of their study. In doing so, researchers are permitted to effectively compare and contrast the mental health outcomes of different groups who engaged with different aspects of social media over a period of time, which may lead to positive advancements in the navigation of the relationship between mental health and social media.

By addressing implications and future directions, stakeholders can advance towards a more thorough understanding of the effect of social media on adolescent mental health and develop more effective strategies to offer support to young people in the present digital age.

In a final note, on 8 November 2024, the Government of Australia banned the use of social media for individuals under 16 years of age, citing adverse health risks associated with over-exposure to social media in young people (Government of Australia, 2024). This has been met with overall international governmental approval but much discussion amongst experts in this field. Such developments underscore the importance and need for clear analysis of evidence in this context. The bottom line from this review of reviews is that social media exposure may have positive or negative effects, but context is important, and we do not have a clear understanding of determinants of the net impact of social media on youth and adolescent mental health.

## Figures and Tables

**Table 1:** Data extracted from each article.

Author/Year	Duration	Total # of Articles	Review Type	Aim of Review	Participants’ Characteristics/Sample Size	Bias Assessment	Social Media Type	Key Findings and Mental Health Implications	Recommendations
Haddad et al. (2021)	Articles published between the beginning of the COVID-19 pandemic and 3 January 2021	6	Multinational review	What is the relationship between social media use and college student mental health during the COVID-19 pandemic?	College students	-	Not specified	Mixed study results; negative, positive, and null effects on mental health.	Explore the following alternate research questions: Do stressors related to disasters become a mediating factor that was not considered prior to the COVID-19 pandemic? Or do our new findings seamlessly integrate so that using social media to relieve boredom leads to more anxiety in the setting of high COVID-19 stressors? Strategies to mitigate the negative effects of social media on mental health during the pandemic include the following: Optimism, mindfulness, and cognitive reappraisal. Avoiding dialectical thinking.
Damodar et al. (2022)	-	23	Systematic review	To evaluate the influence of social media use on adolescent anxiety and depression	Adolescents	-	Facebook, Instagram, Snapchat, Twitter, Myspace, and others	Depression (82.6%) and anxiety (78.3%) were positively associated with social media use in the majority of articles. A dose–response relationship between screen time and increased depression and anxiety symptoms was observed in 13.0% of articles.	Mental health interventions for adolescents were identified as follows: - Screen time restrictions. - Social support. - Education among caregivers concerning nonconfrontational approaches to addressing social media use among their children.
Draženović et al. (2023)	-	13	Review (good)	To assess the impact of S.M. use during the first year of the COVID-19 pandemic on the M.H. of adolescents and students	Adolescents and students, 11,975	The risk of bias in 85% (11/13) of the included studies was classified as low	Not specified	Anxiety, depression, and stress were the most commonly observed mental health outcomes of social media use in a majority of studies. Active and increased social media use was paralleled increased depression, anxiety, and stress symptoms. Increased COVID-19 stress led to an increased risk of addictive social media use. In total, 2/13 studies observed positive influences of social media on mental health, including increased periods of sleep and increased support. Adolescents who spent more time engaging with social media had an increased likelihood of experiencing moderate to extreme symptoms of anxiety.	Longitudinal studies are recommended to observe the influence of different types of S.M. behaviors and activities.
Cunningham et al. (2021)	-	62	Meta-analysis	To examine the quantitative associations of depression symptoms to social networking site (S.N.S) use	Adolescents	-	Not specified	Increased social networking site use paralleled increased depression symptoms. Problematic social networking site use was the primary construct defining the relationship between S.N.S. use and depression, even more than time spent engaging with S.N.S.s or general intensity of S.N.S. use.	Targeted intervention strategies to reduce the negative mental health impact of S.N.S. use would be cost effective and beneficial to individuals engaging in problematic or addictive use of S.N.S.
Liu et al. (2022)	-	26	Meta-analysis	To investigate the association between time spent on social media (TSSM) and depression	55,340 adolescents	Mildly significant based on Egger’s linear regression test	Not specified	A 13% increase was found in depression for each hour increase in social media engagement in adolescents. TSSM is linearly related to depression in a dose–response and gender-dependent manner. The observed association was stronger for adolescent girls than boys.	- To lessen the risk of depression, particularly for adolescent girls, there should be prevention efforts to target a more comprehensive understanding of the effects of time spent on social media.
Zhu et al. (2023)	January 2012 to March 2020	29	Review (good)	To analyze the existing body of work on the relationship between depression and social media use in the information system field	Adolescents	-	Not specified	Social media exposes individuals to prominent qualities of information, which has the potential to augment their cognitive burden and fatigue, thereby making them more susceptible to depression. Social media was identified to have a significant negative impact on depression in some articles. However, other researchers could not identify a significant effect of social media on depression.	-
Keles et al. (2020)	-	13	Systematic review	To synthesize evidence on the influence of social media use on depression, anxiety, and psychological distress in adolescents	Adolescents	Present	Not specified	Mixed findings; some found no relationship between the frequency of social media use and depressive symptoms, while others found that social media use that exceeded 2 h was associated with psychological distress. Others found that there was a positive relationship between social media use, depression, and anxiety. Most authors noted that the relationship was too complex for straightforward statements.	-
Santos et al. (2023)	2013–2023	50	Systematic review	To understand the association between screen time and adolescents’ mental health	Adolescents	-	Not specified	More time spent on social media was associated with a higher prevalence of depression. Social media use had a median negative association with wellbeing. The use of social media negatively impacted mental health but was not significantly associated with self-harm or depression among boys.	- Studies should be conducted to acquire an understanding of adolescents’ motivation to engage with screen devices. - Longitudinal studies that illuminate factors like sleep, physical activity, and socioeconomic status will also be essential in establishing mediators of associations between screen time interactions and mental health in adolescents.
Marciano et al. (2022)	Studies up to September 2021	30	Meta-analysis and systematic review	To study the link between mental health and digital media use in adolescents during COVID-19	Adolescents (median: 760, ranging from 102 to 13,525)	Bias may be present because of the emphasis on the detrimental effects of screen time and social media use, including addictive use; there were limited studies that focused on a positive conceptualization of mental wellbeing	Instagram, Snapchat, TikTok, and others	Not all types of digital media use had adverse mental health outcomes in adolescents. Particularly, one-to-one communication, self-disclosure in the context of mutual online friendship, and positive and humorous online experiences alleviated feelings of loneliness and stress. Generally, increased social media engagement and addiction are related to increased ill-being (symptoms of depression, anxiety, mood disorder, and ruminative thoughts).	- Increased focus should be placed on the positive aspect of mental health for future research. - In future studies, emphasis should be placed on the type and quality of (social) media use. - Researchers should implement increased time ranges during longitudinal studies.
Khalaf et al. (2023)	1 January 2000 and 30 May 2023	11	Systematic review	To identify studies on the influence of technology, primarily social media, on adolescents’ and young adults’ psychosocial functioning, health, and wellbeing	Adolescents and young adults	-	Not specified	Modest but statistically significant association between social media use and depression symptoms. Increased levels of social media engagement were paralleled by poorer mental health outcomes.	- More research is required on social media and mental health to make definitive recommendations for parents, educators, or institutions.
Popat and Tarrant (2023)	January 2014 and December 2020	24	Qualitative literature review	To highlight how social media use can contribute to poor mental health	Adolescents	Minimized bias; however, it is acknowledged that the variability between studies could result in an uneven influence on the conclusions made	Not specified	Social media can positively affect adolescent wellbeing by fostering connection and support through discussion forums. Social media can be harmful in that it can lead to poor mental health through cyberbullying, body comparison, fear of judgment, addiction, etc.	- Looking forward, educational interventions and social media alterations could help buffer against adverse effects.
Schønning et al. (2020)	2015–2020	79	Scoping review	To present a scoping review of the published literature in the research field of social media use and its association with mental health and wellbeing among adolescents	Adolescents	-	Facebook (39%) was the most studied social network site of specified social media	The majority of studies established a correlation between regular use of social media and poorer mental health and wellbeing (e.g., depression, anxiety, alcohol use, psychiatric problems, suicidal behavior, and eating disorders).	- Real-time tracking of actual use and modes of use of social media is encouraged in order to ensure increased accuracy of the different aspects of social media.
Shannon et al. (2022)	-	18	Systematic review and meta-analysis	To systematically examine problematic social media use in youth and its association with symptoms of depression, anxiety, and stress	Adolescents and young adults, 9269 participants	The funnel plot shows slight asymmetry, suggesting slight publication bias; however, Egger’s test for small-study effects was not significant	Not specific	There is evidence for moderate but statistically significant correlations between problematic social media use and depression, anxiety, and stress. Anxiety was observed to have the strongest correlation.	- Research should be conducted to clarify how social media can be used without taking a toll on the mental health of users. - Future studies analyzing the relationship between problematic social media use and mental health outcomes should provide more detailed information on how participants are using various platforms.
Arias-de la Torre et al. (2020)	Information published up to June 2019	7	Umbrella review	To synthesize the current evidence on the association between mobile technologies and social media (MTSM) use and the development or prevention of depressive disorders in adolescents	Adolescents, 5582–46,015 participants	-	Not specific	Increased social comparison and personal involvement during the use of MTSM may be associated with the development of depressive symptomatology. There was a positive and significant relationship between the usage of some dimensions of social media and the presence of depressive symptoms among adolescents. MTSM may also encourage social support and even become a point of assistance for individuals with depression.	- Longitudinal research should be conducted that focuses on specific factors, such as family environment and specific depressive symptoms. - This would be beneficial in potentially preventing the development of depression in MTSM users.
Sedgwick et al. (2019)	2012 and 2018	9	Meta-analysis	To review the relationship between the potential for both detrimental and supportive influences of social media/internet use on suicidal behavior	Adolescents, 346,416	Ascertainment of the exposures and outcomes within each article showed a low risk of bias, but there was significant heterogeneity between studies	Not specific	There were attempts in seven studies, and there was an independent and direct association found between heavy social media/internet use and increased risk of suicide. Two studies found that an amount of social media/internet use, compared to no use, may be associated with fewer suicide attempts. No studies exist investigating the relationship between social media/internet use and completed suicide.	- Research should be conducted to determine which young people access different levels of web content and whether access to the Darknet is associated with more violent or effective means of suicide, which could be important areas of future research.
Sarmiento et al. (2020)	2000–2017	36	Systematic narrative review	To evaluate associations between social media use and the internalizing symptoms of depression, anxiety, and loneliness among 12–18-year-olds	Adolescents	-	Not specific	Studies relating social media to depressive symptoms were more frequently identified than those concerning other internalizing conditions. Studies identified a significant association between the use of social media and symptoms of depression. However, this conclusion is too simplistic. To specify, it is evident that what adolescents do online (e.g., active vs. passive use), under what conditions (e.g., when experiencing stress), and at what time (e.g., before bed) matters. The gender more affected depends on the particular aspect of social media use that investigators measure. Most (88%) of the 24 correlational studies found significant relationships between adolescents’ social media use and feelings of loneliness.	- Heightened attention should be placed on the conceptualization and operationalization of social media and internalizing symptoms. - A movement towards integrated longitudinal and experimental designs would be beneficial.
Thorell et al. (2022)	2011–2021	28	Systematic literature review	To conduct a systematic review of longitudinal studies published within the past 10 years and examine the link between ADHD symptoms and digital media use	Children and adolescents, >66,000	-	Not specified	Associations of social media use with ADHD appeared stronger for longitudinal studies investigating the problematic use of digital media in contrast to those focusing on screen time. Associations were not strongly related to either the age or sex of the child.	- The performance of both moderation and mediation analyses should be an important avenue for future longitudinal research, especially in identifying subgroups as well as underlying factors that can better explain the association between digital media use and ADHD symptom levels.
Prasad et al. (2023)	-	77	A review (good)	The objective of the review is to explore if there is a relationship between social media and the development of two disorders (depression and anxiety) among youth	Youth	-	Not specified	Specific patterns of social media engagement were associated with poor mental health outcomes in youth rather than solely frequency or duration of use. Passive S.M. use among young adults led to poor outcomes in adolescents.	- Future studies should evaluate parents’/guardians’ and pediatricians’ perceptions of children’s social media behaviors and online social engagement more comprehensively, as this may further fortify the understanding of social media and its impact on adolescents.
Ryding and Kuss (2020)	-	40	Systematic review	To identify S.N.S. usage and patterns, S.N.S. features, and mediating factors contributing to body image dissatisfaction	Adolescents	-	Facebook, Instagram, and others	The majority of findings suggested that frequent use of S.N.S. is associated with heightened body image concern and dissatisfaction, with many studies in particular focusing on Facebook. Generally, the type of engagement on S.N.S., including, in particular, passive use and appearance-focused S.N.S. use, significantly paralleled greater body image dissatisfaction. It is suggested that frequent S.N.S. comparisons may result in the onset of subthreshold BDD, triggering increased S.N.S. usage and, therefore, the maintenance of BDD symptoms.	- Development of body image measures that are specific to differential S.N.S. environments would be helpful for future research and would facilitate more comprehensive insight into the specific features of S.N.S. that lead to body image disturbance.
Lee et al. (2022)	March 2020 to December 20, 2020	14	Meta-analysis	To summarize the association between time spent on social media platforms during COVID-19 quarantine and mental health outcomes (i.e., anxiety and depression)	Young adults	No significant publication bias	Facebook, Twitter, Instagram, and others	Increased time spent using social media platforms was associated with anxiety symptoms in overall studies. An increase in social media use time was also associated with depressive symptoms. Excessive time spent on social media platforms was associated with an increased likelihood of having symptoms of anxiety and depression.	- Further observation studies with longitudinal design to determine the true effect of social media platforms are required.
Senekal et al. (2023)	2008–2019	20	Systematic review	To review the effects of exposure to social media on the psychosocial development of adolescents	Adolescents	A threshold score of 70% was set to include articles of strong to excellent methodological quality; studies achieving below 70% were excluded to minimize the risk of bias; however, potentially relevant findings may have been excluded	Not specific	Longitudinally, the review found that exposure to cyberbullying is associated with increased depression but not anxiety. This may be explained by the general progression of the disorder, that is, ‘depressive reactions are preceded by prodromal periods of chronic anxiety’. Furthermore, the negative impact of problematic use, in terms of decreased academic performance and a negative impact on social relationships, may exacerbate depressive symptoms. It is when adolescents are invested or engrossed in social media, not the frequency of use, that predicts depressed mood and lower self-esteem. A general trend was identified in decreased adolescent wellbeing, with indications of directionality relating to increased uptake of smartphones. Social media is not problematic in itself; whether it is problematic depends on its appropriate use.	- Additional longitudinal research should be conducted on this topic.
Wong et al. (2022)	Between the pandemic years	38	Meta-analysis	To synthesize previous research data in order to gain a holistic understanding of the association between social media and wellbeing, particularly in the present context of COVID-19	-	The negative association between social media usage and wellbeing found in the study might have a higher likelihood of being biased	Not specific	The relationship between social media usage and wellbeing was not significant in the context of COVID-19. The impact of various moderators on the relationship between social media and wellbeing varied. The review found that there was no significant association between social media usage and wellbeing. The tendency for individuals to use social media as a coping mechanism was negatively correlated with heightened levels of wellbeing. This is suggestive of the fact that individuals who have a greater tendency to use social media as a form of coping are more susceptible to experiencing poorer emotional wellbeing than individuals who have a lower tendency to use social media as a form of coping. Overall, the study observed that there was a negative but nonsignificant correlation between social media and wellbeing during pandemic times.	- Future research should conduct more longitudinal studies in order to ascertain the directionality of the relationship between social media usage and wellbeing. - It was found that there was a lack of sufficient data to conduct an analysis on many moderators. This highlights the need for future researchers to look at different moderators when conducting studies on social media and wellbeing.
Chen et al. (2024)	2014–2024	19	Meta-analysis	To examine the associations between screen-based activities and suicidal behaviors among young people	Young people, 43,489	Based on the result of the trim-and-fill method, it appears that potential publication bias is unlikely to significantly influence the interpretation of the results of the meta-analysis	Not specified	The review found that total screen use and cyberbullying victimization are associated with heightened risks of subsequent self-harm and suicidal behavior in young people. Social media use and problematic screen use are significant risk factors for self-harm and suicidal behaviors.	- The study emphasizes the need for further longitudinal studies to be conducted to reveal the association of various types of screen-based activity with self-harm and suicidal behavior. - It is important to investigate the dose–response relationships between screen-based activities and negative mental health outcomes.
Dharejo et al. (2023)	2013–2023	111	Systematic literature review	Examines the link between youths’ usage of social media and their affective W.B. to close the knowledge gap	Adolescents	-	Not specified	The use of social media in moderation and with knowledge has been linked to better health, including social support and connection. There is additional evidence linking excessive consumption of social media and improper internet behavior to poor mental health outcomes, especially in young people who are more susceptible to these behaviors.	- A deeper comprehension of the intricate relationships between social media use and wellbeing can ultimately be attained through thorough research and analysis. This ensures evidence-based tactics to promote positive online conduct and to ensure young people’s emotional wellbeing.
Valkenburg et al. (2022)	2019 to mid 2021	25	Umbrella review	To fill the gap in knowledge on social media use and how it affects adolescent mental health	Adolescents	Many reviews observed an over-reliance on self-report measures of social media use and its outcomes, which may have introduced various biases	Not specified	The meta-analytic evidence presented in the review suggests that S.N.S. use is weakly associated with higher levels of ill-being and, simultaneously, with higher levels of wellbeing. This suggests that ill-being is not necessarily the mere flip-side of wellbeing and vice versa. Rather, both outcomes should be investigated in their own right. Time spent with S.M. explains only 6% of problematic S.M.U.	- More research needs to investigate how S.M.U. can be used to promote mental health among youth. - Longitudinal studies are also required to determine the causal direction of the effects of S.M.U. on mental health.
Mazzeo et al. (2024)	-	-	A review	To review research on social media and adolescent body image and to discuss strategies to reduce risks associated with social media use	Adolescents	-	Instagram, TikTok, and others	Adolescents with a higher number of social media accounts and those who spend more time on social media are more susceptible to engaging in disordered eating behaviors. To specify, image-based platforms, such as Instagram and TikTok, tend to pose a greater risk, as posting and interacting with image-based content appears to have a more detrimental impact on body image than text-based content.	- Interventions, body-positive content, and policy change are potential pathways for harm reduction. - Additional research is required to investigate their effectiveness both over the long term and in more diverse racial, ethnic, and gender groups.
Sharma and Vidal (2023)	2015–2022	70	A scoping literature review	To review the existing literature considering the effects of highly visual social media (HVSM) on the development of eating disorders (E.D.) and disordered eating (D.E.) in the adolescent and young adult (AYA) population	Young adults	May be present	Highly visual social media (Instagram and Snapchat)	A strong relationship was identified between HVSM, E.D., and D.E., with existing gender differences related to the nature of engagement and preferences regarding content. Total time spent on HVSM, obsession with photo editing, and posting with blurred demarcation of real vs. virtual presentations is associated with DE/ED development. HVSM also has the potential to be used as a therapeutic tool for the treatment of DE/ED, as proven in a feasibility study, which analyzed the efficacy of an intervention focused on appearance-related social media use among young adults who are at increased risk of developing an E.D. Time spent on HVSM is substantially and positively associated with measures of symptomatology of E.D., such as body dissatisfaction, and negatively associated with measures of psychological health in both genders. The use of unrestricted and profit-driven S.M. platforms can increase risk for E.D.	- Additional longitudinal and randomized control trials are needed to understand and address discrepancies concerning gender, age, sociocultural factors, and psychosocial individual and family determinants in order to more distinctively define the relationship between HVSM use and DE/ED.
Tibber and Silver (2022)	-	-	A review	To present a trans-diagnostic cognitive–behavioral conceptualization of the positive and negative roles of social media use in adolescence, with a focus on how it interacts with common mental health difficulties	Adolescents	Not explicitly stated	Facebook and others	There is existing evidence for a weak association between greater usage of social media and poorer mental health, including but not limited to symptoms of depression, anxiety, and general distress. There exists mixed evidence that suggests bidirectional/reciprocal effects; thus, whilst high levels of social media use may negatively impact mental health, poorer mental health may also drive increased social media use.	-
Abi-Jaoude et al. (2020); effects appear to be greatest among girls	-	-	A review	To review the evidence that links smartphone and social media use with mental distress and suicidality among adolescents	Youth	-	Instagram, Facebook, etc.	Social media platforms included normalization of self-harm behavior, discussions about practical issues regarding suicidality, and live depictions of self-harm acts. Simultaneously, there were positive elements, such as providing a sense of community, suggestions for seeking treatment, and advice on stopping self-harm behavior. A multitude of cross-sectional, longitudinal, and empirical studies indicated that smartphone and social media use was linked to an increase in mental distress, self-injurious behavior, and suicidality among youth. A dose–response relationship was observed, and the effects were evidently greatest among girls. Social media content often involves normalization and even promotion of self-harm and suicidality among youth.	- With the backdrop of policy initiatives aimed at addressing the social, environmental, and economic factors that support family wellbeing and foster youth resilience, today’s youth could benefit from proven individual and systemic interventions. This can help them navigate the challenges brought about by the use of smartphones and social media so they can protect themselves from harm and use social media in a way that safeguards their mental health and wellbeing.
Cataldo et al. (2021)	2010–2021	-	A narrative review	To provide an overview of the evidence linking fitspiration trends on social media to mental health disturbances	Young adults	-	Facebook, Tumblr, Twitter, Pinterest, etc.	Fitspiration trends subject young people to self-objectification and unsafe behaviors, thereby enhancing their susceptibility to developing psychopathological symptoms as well as clinically significant conditions (e.g., eating disorders, mood and anxiety disturbances, substance misuse disorders, BDD, or M.D. It also contributes to the fortification of underlying dysfunctional beliefs or attitudes towards one’s own body image).	- Additional research is required to determine the extent of the harm to young people and to develop preventive mental health strategies.
Rathod et al. (2022)	-	84	A review	To summarize the present situation and the correlation between smartphones and mental health	Adolescents and young adults	-	-	Negative attitudes and feelings of fear due to smartphone usage are associated with a heightened risk of depression and anxiety. The majority of studies on this issue revealed a relationship between depression and smartphone usage. Children who are subject to cyberbullying are more likely to experience psychosomatic issues, such as chronic headaches, sleep issues, anxiety, and despair. The review found that smartphone usage predisposed adolescents to mental health issues, insomnia, cyberbullying, anxiety, depression, obesity, false prestige, self-control issues, physiological stress, vision problems, mind-wandering, attention deficit–hyperactivity disorder (ADHD), and obsessive–compulsive disorder (O.C.D.).	-
Vidal et al. (2020)	2011–2019	42	Scoping review	To examine the bi-directional association between the use of S.M., specifically social networking sites (S.N.S.), and depression and suicidality among adolescents	Adolescents	-	Not specified	Most studies identified a positive correlation between time spent on S.N.S. and higher levels of depression. Blomfield-Neira and Barber identified an association between adolescents with a social media profile and depressed mood; however, they found no correlation between social media, frequency of use, and depressed mood. Alternately, it was an investment in social media (a measure of the importance of social media to an adolescent) that was linked to poorer adjustment, lower self-esteem, and depressed mood. Moderate use of social media (a stable trend in the time spent on S.M. during adolescence and into early adulthood that did not interfere with functioning) was associated with improved emotional self-regulation. Some aspects of social media have positively influenced adolescents’ wellbeing, such as their ability to acquire diverse friendships and easily access support.	- Research in this field would benefit from the use of the following: Longitudinal design objectives and timely measures of S.M. use to research the mechanisms of the association between S.M. use and depression and suicidality. Research in clinical populations to inform clinical practice.
Yue and Rich (2023)	2018–2023	-	Review	To delve into the intricate relationship between social media and adolescent mental health	Adolescents	Not mentioned	Not specified	A multifaceted relationship exists between social media use and adolescent mental health. The effects are molded by how, why, when, and by whom social media is used. The majority of studies suggest a positive correlation between prolonged social media usage and elevated symptoms of depression among adolescents. The consequences of social media use for adolescent mental health are multifaceted and defy monolithic classification. It is essential to recognize the intermediary variables that shape these observed interactions in order to unravel this complex interplay.	- Research must consider design features of the various social media ‘spaces’, as well as the psychological states of users that mold the relationship in order to acquire a more comprehensive understanding.
Sala et al. (2024)	January 2015–April 2023	24	Umbrella review	To analyze the risks and opportunities for adolescents’ mental health and wellbeing associated with social media	Adolescents	The authors observe an over-representation of white adolescent students living in the global North, the so-called WEIRD (Western, educated, industrialized, rich, and democratic) bias	Not specified	The impact of social media use is dependent on personal characteristics, usage type, and platform design. The correlation between time spent on S.M. and depressive symptoms is statistically significant for both sexes and even stronger for girls. Some studies found that moderate use of S.M. (approximately less than two hours per day) was associated with greater wellbeing, social support, improved social relationships, and participation in social and political life. Passive consumption of S.M. is associated with heightened levels of social comparison, decreased perceived social support, envy, and depressive mood. On the other hand, active use is associated with increased wellbeing and life satisfaction and not associated with later depressed mood. It was noted that providing and receiving online social support is associated with greater general mental health, life satisfaction, and wellbeing. Social media access may be an avenue for mood regulation and stress management and to escape boredom or emotions like anger, loneliness, sadness, or anxiety. However, when this results in an augmented amount of time spent engaging with social media, it can represent a risk for diminished wellbeing. The associations between S.M.U. and (i) depressive and anxiety symptoms, (ii) problematic use and addiction, (iii) eating behaviors and body image concerns, and (iv) deliberate self-harm and suicidality are among the identified risks to which adolescents are subjected. There are several intervening factors mediating the relationships between adolescents’ social media use and mental health. The review identified demographic and psycho-socio characteristics, the specific type of use of S.M., and the platforms’ content and design.	Need for research considering specific groups of populations and specific platforms’ designs and mechanisms to provide more fine-grained evidence of the potential impact of S.M.U. on adolescents’ wellbeing.
Suhag and Rauniyar (2024)	-	-	Review	Investigates the link between social media usage and the development of binge eating disorder patterns and undesirable body image judgments	Adolescents	-	Instagram and TikTok, Snapchat, Facebook, and others	Social media platforms that place major emphasis on physical attractiveness contribute to heightened levels of stress and eating disorders. Spending more time online, especially in self image and eating-related activities, was associated with less satisfaction with one’s body image, as well as unhealthy eating habits.	- It is essential for researchers and professionals to explore how the internet can foster a positive digital environment and be a source of empowerment for young adolescents.
Primack et al. (2022)	Media published by 1 May 2021	-	Review	To broadly summarize how S.M. interfaces with both psychosocial development and mental health conditions among young adults	Youth	-	-	Increased social media use may positively impact adolescents’ affective and cognitive empathy and broaden their ability to communicate their feelings and appreciate the feelings of others. S.M. may offer opportunities to augment traditional mental health treatment, and it may also facilitate forming connections among people with health disorders, such as anxiety, depression, schizophrenia, and autism spectrum disorders. A study found consistent, linear associations between the quartile of S.M. use and the degree of depression based on patient self-reported outcomes. In contrast with individuals in the lowest quartile, individuals in the highest quartile of social media application visits per week had significantly heightened odds of depression. In one study, S.M. use at baseline was strongly associated with the development of depression among nondepressed individuals over the subsequent 6 months. lso, an online survey conducted in Norway among 23,533 individuals aged 16 years and older, found a positive and significant relationship between symptoms of anxiety, obsessive–compulsive disorder, and potentially addictive use of S.M. Researchers in another study identified significant associations between problematic use of Facebook, social anxiety, and the need for social assurance. It has also been identified that pro-anorexia S.M. content can be influential with regard to male body image issues. Large cross-sectional epidemiologic studies have found that heightened S.M. engagement is linearly related to the prevalence of mental health concerns, such as depression, anxiety, and sleep disturbance.	- Many cross-sectional studies are present; however, there is a need for longitudinal studies to be published. - There has been a strong focus on community-based populations; however, it would be beneficial for future work to involve clinical populations to facilitate discoveries with clinically relevant conclusions. For example, the discovery of associations between S.M. use and mental disorders suggests that it may be valuable for clinicians to assess S.M. use among depressed individuals to identify maladaptive patterns of use, which may be contributing to mood dysregulation.
Ivie et al. (2020)	1 January 2017 2020	12	Meta-analysis	The authors conducted a meta-analysis of studies that measured the association between social media use specifically and depressive symptoms among early to mid adolescents	Adolescents, 92,371 participants	There is an absence of small study bias (and, arguably, little publication bias)	-	There was a small but significant positive correlation between adolescent social media use and depressive symptoms. High heterogeneity indicated significant variation between studies. This suggests that other factors are likely to act as significant moderators of the relationship.	- We suggest that future research should focus on understanding which types of use may be harmful (or helpful) to mental health rather than focusing on overall use measures.
Biernesser et al. (2020)	25 June 25 2014 to 20 May 20 2018; subsequently updated for articles published from 21 May 2018 to 24 September 2019	38	Systematized narrative review	To review social media use and youths’ deliberate self-harm for mental health services professionals	Youth	Potentially present	-	The two most recent systematic reviews identified that subjection to and expression of deliberate self-harm content via social media were associated with the adoration and normalization of self-harm behavior, as well as a multitude of other negative behaviors that heightened the risk of future deliberate self-harm. Though social isolation was identified as a concern, the potential for social media to reduce isolation and be a positive factor in fostering social connectedness was discussed as a potential benefit.	- Further research is required to acquire a thorough comprehension of the contribution of social media use to youth D.S.H., especially among youth most vulnerable to suicidal risk.
McCrory et al. (2020)	2010 to March 2019	25	Scoping review	To conduct a scoping review of the current literature on the relationship between highly visual social media and young people’s mental health	Young people (adolescents)	-	HVSM—e.g., Snapchat, Facebook, and Instagram	Three studies (20%) identified that increased social media usage in terms of time was beneficial to wellbeing. Thirteen studies (87%) found a negative impact on individuals. Four studies (27%) identified that no relationship exists. All studies investigating the alteration of photos navigated the relationship with body image/body dissatisfaction and identified negative connections.	- Preceding the development of interventions to improve the mental health of young people when highly visual social media has produced negative outcomes, the nature of the relationships using methodologies that can explore and delve deeper into the impact of HVSM should be explored to a greater extent.
Piteo and Ward (2020)	January 2005–March 2019	19	Systematic review	To examine the relationship between S.N.S. use and depressive and anxiety symptoms in the child and adolescent population	Adolescents and children	Likely present due to the self-report questionnaires used	Facebook, Instagram, etc.	Elevated hours spent on or frequency of S.N.S. use, as well as problematic and addictive behavior on social networking sites, were significantly related to heightened levels of depressive symptoms. Two cross-sectional studies identified that increased time spent on or frequency of social networking site use was associated with increased levels of investment in S.N.S. and anxiety symptoms.	- Future studies should explore the multiple conditions through which S.N.S. may either hinder or fortify the development of emotional regulation in young people.
Moss et al. (2023)	-	15	Scoping review	To investigate the extent to which Instagram use impacts the mental health of its adolescent users; specifically, to identify if there is a relationship between time spent on Instagram and engagement in deliberate self-harm	Adolescents	-	Instagram and other forms of social media	There is a correlation between time spent on Instagram and adolescent engagement in deliberate self-harm activities.	Further research should be conducted to distinguish whether there is a difference between older and younger adolescents when using social media and whether there is cause for concern across the various other social media platforms or Instagram alone. Further restrictions must be implemented to decrease the number of adolescents exposed to and consequently engaging in D.S.H. activities.
Vincente-Benito and del Valle Ramirez-Duran (2023)	2015–April 2020	21	Systematic review	To analyze social media’s impact on body image and wellbeing among adolescents and young adults	Adolescents and young adults	Bias may be present due to self-report techniques used to measure mental health outcomes, as well as the lack of delimitation concerning the hours of use of these platforms	Not specified	Misuse or intensive use of social media was associated low self-esteem, risky behaviors, and eating disorders. The relationship is fortified by moderating factors, such as comparisons of physical appearances. The impact of social media on body image and wellbeing in adolescents and young adults is mostly detrimental.	- It is recommended that the influence of social media be analyzed from a gender-based perspective. - It is additionally essential to analyze the effects of social media on children.
Rafiq and Linden (2024)	2012–January 2023	41	Scoping review	To investigate the relationship between S.N.S. use and self-concept, which has not yet been explored in depth among the postsecondary population	Adolescents	Self-report measures used in the majority of studies may have led to skewed results owing to social desirability bias or exaggerated responses	Mostly Facebook, Twitter, and Instagram	The review revealed that a majority of students engage in upward appearance-based comparisons with their friends or peers on S.N.S., as they consider them to be appropriate competition. This results in decreased self-worth based on physical appearance. Engaging in online upward comparisons based on competence or academics results in a diminished mental state and self-esteem among postsecondary students. Finally, although S.N.S. promotes social connectedness, chronic use can result in social disengagement owing to upward comparisons based on S.N.S. follower count.	Future research should explore how S.N.S. has influenced upward academic comparisons among university students in more recent years.

## Data Availability

No new data were created or analyzed in this study.

## References

[B1-behavsci-15-00574] Abi-Jaoude E., Naylor K. T., Pignatiello A. (2020). Smartphones, social media use and youth mental health. CMAJ.

[B2-behavsci-15-00574] Arias-de la Torre J., Puigdomenech E., García X., Valderas J. M., Eiroa-Orosa F. J., Fernández-Villa T., Molina A. J., Martín V., Serrano-Blanco A., Alonso J., Espallargues M. (2020). Relationship between depression and the use of mobile technologies and social media among adolescents: Umbrella review. Journal of Medical Internet Research.

[B3-behavsci-15-00574] Arksey H., O’Malley L. (2005). Scoping studies: Towards a methodological framework. International Journal of Social Research Methodology.

[B4-behavsci-15-00574] Biernesser C., Sewall C. J. R., Brent D., Bear T., Mair C., Trauth J. (2020). Social media use and deliberate self-harm among youth: A systematized narrative review. Children and Youth Services Review.

[B5-behavsci-15-00574] Blomfield neira C. J., Barber B. L. (2014). Social networking site use: Linked to adolescents’ social self-concept, self-esteem, and depressed mood. Australian Journal of Psychology.

[B6-behavsci-15-00574] Bottaro R., Faraci P. (2022). The use of social networking sites and its impact on adolescents’ emotional well-being: A scoping review. Current Addiction Reports.

[B7-behavsci-15-00574] Bozzola E., Spina G., Agostiniani R., Barni S., Russo R., Scarpato E., Di Mauro A., Di Stefano A. V., Caruso C., Corsello G., Staiano A. (2022). The use of social media in children and adolescents: Scoping review on the potential risks. International Journal of Environmental Research and Public Health.

[B8-behavsci-15-00574] Cataldo I., De Luca I., Giorgetti V., Cicconcelli D., Bersani F. S., Imperatori C., Abdi S., Negri A., Esposito G., Corazza O. (2021). Fitspiration on social media: Body-image and other psychopathological risks among young adults. A narrative review. Emerging Trends in Drugs, Addictions, and Health.

[B9-behavsci-15-00574] Chen Z., Liao X., Yang J., Tian Y., Peng K., Liu X., Li Y. (2024). Association of screen-based activities and risk of self-harm and suicidal behaviors among young people: A systematic review and meta-analysis of longitudinal studies. Psychiatry Research.

[B10-behavsci-15-00574] Course-Choi J., Hammond L. (2021). Social Media use and adolescent well-being: A narrative review of longitudinal studies. Cyberpsychology, Behavior, and Social Networking.

[B11-behavsci-15-00574] Cunningham S., Hudson C. C., Harkness K. (2021). Social media and depression symptoms: A meta-analysis. Research on Child and Adolescent Psychopathology.

[B12-behavsci-15-00574] Damodar S., Lokemoen C., Gurusamy V., Takhi M., Bishev D., Parrill A., Deviney M., Person U., Korie I., Branch R. (2022). Trending: A systematic review of social media use’s influence on adolescent anxiety and depression. Adolescent Psychiatry.

[B13-behavsci-15-00574] Davila J., Hershenberg R., Feinstein B. A., Gorman K., Bhatia V., Starr L. R. (2012). Frequency and quality of social networking among young adults: Associations with depressive symptoms, rumination, and corumination. Psychology of Popular Media Culture.

[B14-behavsci-15-00574] Dharejo N., Alivi M. A., Rahamad M. S., Jiaqing X., Brony M. (2023). Effects of social media use on adolescent psychological well-being: A Systematic literature review. International Journal of Interactive Mobile Technologies.

[B15-behavsci-15-00574] Draženović M., Vukušić Rukavina T., Machala Poplašen L. (2023). Impact of social media use on mental health within adolescent and student populations during COVID-19 pandemic: Review. International Journal of Environmental Research and Public Health.

[B16-behavsci-15-00574] Frison E., Eggermont S. (2015). The impact of daily stress on adolescents’ depressed mood: The role of social support seeking through Facebook. Computers in Human Behavior.

[B17-behavsci-15-00574] Frison E., Eggermont S. (2016). Exploring the Relationships between different types of facebook use, perceived online social support, and adolescents’ depressed mood. Social Science Computer Review.

[B18-behavsci-15-00574] Government of Australia (2024). Minimum age for social media access to protect Australian kids.

[B19-behavsci-15-00574] Haddad J. M., Macenski C., Mosier-Mills A., Hibara A., Kester K., Schneider M., Conrad R. C., Liu C. H. (2021). The impact of social media on college mental health during the COVID-19 pandemic: A multinational review of the existing literature. Current Psychiatry Reports.

[B20-behavsci-15-00574] Ivie E. J., Pettitt A., Moses L. J., Allen N. B. (2020). A meta-analysis of the association between adolescent social media use and depressive symptoms. Journal of Affective Disorders.

[B21-behavsci-15-00574] Jiotsa B., Naccache B., Duval M., Rocher B., Grall-Bronnec M. (2021). Social media use and body image disorders: Association between frequency of comparing one’s own physical appearance to that of people being followed on social media and body dissatisfaction and drive for thinness. International Journal of Environmental Research and Public Health.

[B22-behavsci-15-00574] Keles B., McCrae N., Grealish A. (2020). A systematic review: The influence of social media on depression, anxiety and psychological distress in adolescents. International Journal of Adolescence and Youth.

[B23-behavsci-15-00574] Khalaf A. M., Alubied A. A., Rifaey A. A. (2023). The impact of social media on the mental health of adolescents and young adults: A systematic review. Cureus.

[B24-behavsci-15-00574] Kieling C., Baker-Henningham H., Belfer M., Conti G., Ertem I., Omigbodun O., Srinath S., Ulkuer N., Rahman A. (2011). Child and adolescent mental health worldwide: Evidence for action. Lancet.

[B25-behavsci-15-00574] Kucharczuk A. J., Oliver T. L., Dowdell E. B. (2022). Social media’s influence on adolescents’ food choices: A mixed studies systematic literature review. Appetite.

[B26-behavsci-15-00574] Lee Y., Jeon Y. J., Kang S., Shin J. I., Jung Y. C., Jung S. J. (2022). Social media use and mental health during the COVID-19 pandemic in young adults: A meta-analysis of 14 cross-sectional studies. BMC Public Health.

[B27-behavsci-15-00574] Lin L. Y., Sidani J. E., Shensa A., Radovic A., Miller E., Colditz J. B., Hoffman B. L., Giles L. M., Primack B. A. (2016). Association between social media use and depression among U.S. Young adults. Depress Anxiety.

[B28-behavsci-15-00574] Liu M., Kamper-DeMarco K. E., Zhang J., Xiao J., Dong D., Xue P. (2022). Time spent on social media and risk of depression in adolescents: A dose-response meta-analysis. International Journal of Environmental Research and Public Health.

[B29-behavsci-15-00574] Marciano L., Ostroumova M., Schulz P. J., Camerini A.-L. (2022). Digital media use and adolescents’ mental health during the COVID-19 pandemic: A systematic review and meta-analysis. Frontiers in Public Health.

[B30-behavsci-15-00574] Mazzeo S. E., Weinstock M., Vashro T. N., Henning T., Derrigo K. (2024). Mitigating harms of social media for adolescent body image and eating disorders: A review. Psychology Research and Behavior Management.

[B31-behavsci-15-00574] McCashin D., Murphy C. M. (2023). Using TikTok for public and youth mental health—A systematic review and content analysis. Clin Child Psychol Psychiatry.

[B32-behavsci-15-00574] McCrory A., Best P., Maddock A. (2020). The relationship between highly visual social media and young people’s mental health: A scoping review. Children and Youth Services Review.

[B33-behavsci-15-00574] Moss C., Wibberley C., Witham G. (2023). Assessing the impact of Instagram use and deliberate self-harm in adolescents: A scoping review. International Journal of Mental Health Nursing.

[B34-behavsci-15-00574] O’Reilly M. (2020). Social media and adolescent mental health: The good, the bad and the ugly. Journal of Mental Health.

[B35-behavsci-15-00574] Piteo E. M., Ward K. (2020). Review: Social networking sites and associations with depressive and anxiety symptoms in children and adolescents—A systematic review. Child and Adolescent Mental Health.

[B36-behavsci-15-00574] Plackett R., Sheringham J., Dykxhoorn J. (2023). The longitudinal impact of social media use on UK adolescents’ mental health: Longitudinal observational study. Journal of Medical Internet Research.

[B37-behavsci-15-00574] Popat A., Tarrant C. (2023). Exploring adolescents’ perspectives on social media and mental health and well-being—A qualitative literature review. Clinical Child Psychology and Psychiatry.

[B38-behavsci-15-00574] Prasad S., Souabni S. A., Anugwom G., Aneni K., Anand A., Urhi A., Obi-Azuike C., Gibson T., Khan A., Oladunjoye F. (2023). Anxiety and depression amongst youth as adverse effects of using social media: A Review. Annals of Medicine and Surgery.

[B39-behavsci-15-00574] Primack B. A., Perryman K. L., Crofford R. A., Escobar-Viera C. G. (2022). Social media as it interfaces with psychosocial development and mental illness in transitional-age youth. Child and Adolescent Psychiatric Clinics of North America.

[B40-behavsci-15-00574] Rafiq A., Linden B. (2024). Social media and self-concept among postsecondary students: A scoping review. Cyberpsychology, Behavior and Social Networking.

[B41-behavsci-15-00574] Rathod A. S., Ingole A., Gaidhane A., Choudhari S. G. (2022). Psychological morbidities associated with excessive usage of smartphones among adolescents and young adults: A review. Cureus.

[B42-behavsci-15-00574] Ryding F. C., Kuss D. J. (2020). The use of social networking sites, body image dissatisfaction, and body dysmorphic disorder: A systematic review of psychological research. Psychology of Popular Media.

[B43-behavsci-15-00574] Sala A., Porcaro L., Gomez E. (2024). Social media use and adolescents’ mental health and well-being: An umbrella review. Computers in Human Behavior Reports.

[B44-behavsci-15-00574] Santos R. M. S., Mendes C. G., Sen Bressani G. Y., de Alcantara Ventura S., de Almeida Nogueira Y. J., de Miranda D. M., Romano-Silva M. A. (2023). The associations between screen time and mental health in adolescents: A systematic review. BMC Psychology.

[B45-behavsci-15-00574] Sarmiento I. G., Olson C., Yeo G., Chen Y. A., Toma C. L., Brown B. B., Bellmore A., Mares M.-L. (2020). How does social media use relate to adolescents’ internalizing symptoms? Conclusions from a systematic narrative review. Adolescent Research Review.

[B46-behavsci-15-00574] Schønning V., Hjetland G. J., Aarø L. E., Skogen J. C. (2020). Social media use and mental health and well-being among adolescents—A scoping review. Frontiers in Psychology.

[B47-behavsci-15-00574] Sedgwick R., Epstein S., Dutta R., Ougrin D. (2019). Social media, internet use and suicide attempts in adolescents. Current Opinion in Psychiatry.

[B48-behavsci-15-00574] Senekal J. S., Groenewald G. R., Wolfaardt L., Jansen C., Williams K. (2023). Social media and adolescent psychosocial development: A systematic review. South African Journal of Psychology.

[B49-behavsci-15-00574] Shannon H., Bush K., Villeneuve P. J., Hellemans K. G., Guimond S. (2022). Problematic social media use in adolescents and young adults: Systematic review and meta-analysis. JMIR Mental Health.

[B50-behavsci-15-00574] Sharma A., Vidal C. (2023). A scoping literature review of the associations between highly visual social media use and eating disorders and disordered eating: A changing landscape. Journal of Eating Disorders.

[B51-behavsci-15-00574] Somerville L. H. (2013). Special issue on the teenage brain: Sensitivity to social evaluation. Current Directions in Psychological Science.

[B52-behavsci-15-00574] Suhag K., Rauniyar S. (2024). Social media effects regarding eating disorders and body image in young adolescents. Cureus.

[B53-behavsci-15-00574] Thorell L. B., Buren J., Wiman J. S., Sandberg D., Nutley S. B. (2022). Longitudinal associations between digital media use and ADHD symptoms in children and adolescents: A systematic literature review. European Child & Adolescent Psychiatry.

[B54-behavsci-15-00574] Tibber M. S., Silver E. (2022). A trans-diagnostic cognitive behavioural conceptualisation of the positive and negative roles of social media use in adolescents’ mental health and wellbeing. Cognitive Behaviour Therapist.

[B55-behavsci-15-00574] Ulvi O., Karamehic-Muratovic A., Baghbanzadeh M., Bashir A., Smith J., Haque U. (2022). Social media use and mental health: A global analysis. Epidemiologia.

[B56-behavsci-15-00574] Valkenburg P. M., Meier A., Beyens I. (2022). Social media use and its impact on adolescent mental health: An umbrella review of the evidence. Current Opinion in Psychology.

[B57-behavsci-15-00574] Vidal C., Lhaksampa T., Miller L., Platt R. (2020). Social media use and depression in adolescents: A scoping review. International Review of Psychiatry.

[B58-behavsci-15-00574] Vincente-Benito I., del Valle Ramirez-Duran M. (2023). Influence of social media use on body image and well-being among adolescents and young adults: A systematic review. Journal of Psychosocial Nursing and Mental Health Services.

[B59-behavsci-15-00574] Wang Y., Xu J., Xie T. (2022). Social media overload and anxiety among university students during the COVID-19 Omicron wave lockdown: A cross-sectional study in Shanghai, China, 2022. International Journal of Public Health.

[B60-behavsci-15-00574] Weigle P. E., Shafi R. M. A. (2024). Social media and youth mental health. Current Psychiatry Reports.

[B61-behavsci-15-00574] Wong J., Yi P. X., Quek F. Y. X., Lua V. Y. Q., Majeed N. M., Hartanto A. (2022). A four-level meta-analytic review of the relationship between social media and well-being: A fresh perspective in the context of COVID-19. Current Psychology.

[B62-behavsci-15-00574] Xavier M., Baptista H., Mendes J. M., Magalhães P., Caldas-de-Almeida J. M. (2013). Implementing the world mental health survey initiative in portugal—Rationale, design and fieldwork procedures. International Journal of Mental Health Systems.

[B63-behavsci-15-00574] Yoshida Y., Sitas N., Mannetti L., O’Farrell P., Arroyo-Robles G., Berbés-Blázquez M., González-Jiménez D., Nelson V., Niamir A., Harmáčková Z. V. (2024). Beyond academia: A case for reviews of gray literature for science-policy processes and applied research. Environmental Science & Policy.

[B64-behavsci-15-00574] Yue Z., Rich M. (2023). Social media and adolescent mental health. Current Pediatrics Reports.

[B65-behavsci-15-00574] Zhu W., Mou J., Benyoucef M., Kim J., Hong T., Chen S. (2023). Understanding the relationship between social media use and depression: A review of the literature. Online Information Review.

